# General Component Analysis (GCA): A new approach to identify Chinese corporate bond market structures

**DOI:** 10.1371/journal.pone.0199500

**Published:** 2018-07-09

**Authors:** Lei Wang, Yan Yan, Xiaoteng Li, Xiaosong Chen

**Affiliations:** 1 School of Economics and Management, University of Chinese Academy of Sciences, Beijing, China; 2 Key Laboratory of Big Data Mining and Knowledge Management, Chinese Academy of Sciences, Beijing, China; 3 Institute of Theoretical Physics, Chinese Academy of Sciences, Beijing, China; Central South University, CHINA

## Abstract

PCA has been widely used in many fields to detect dominant principle components, but it ignores the information embedded in the remaining components. As a supplement to PCA, we propose the General Component Analysis (GCA). The inverse participation ratios (IPRs) are used to identify the global components (GCs) and localized components (LCs). The mean values of the IPRs derived from the shuffled data are taken as the natural threshold, which is exquisite and novel. In this paper, the Chinese corporate bond market is analyzed as an example. We propose a novel network method to divide time periods based on micro data, which performs better in capturing the time points when the market state switches. As a result, two periods have been obtained. There are two GCs in both periods, which are influenced by terms to maturity and ratings. Besides, there are 382 LCs in Period 1 and 166 LCs in Period 2. In the LC portfolios there are two interesting bond collections which are helpful to understand the thoughts of the investors. One is the supper AAA bond collection which is believed to have implicit governmental guarantees by the investors, and the other is the overcapacity industrial bond collection which is influenced by the supply-side reform led by the Chinese government. GCA is expected to be applied to other complex systems.

## Introduction

Principal component analysis (PCA) is one of the most established methods used in the fields of not only science but also economics [1, 2]. Many attempts have been made to extend the scope of PCA. A recent study proposed a non-stationary principal component analysis based on the detrended cross-correlation and showed that the new method could identify the patterns of data in the case of non-stationarity [1]. Another recent study applied PCA to identify the group dynamics and states of global stock markets [2].

Through the PCA method, the components with the largest eigenvalues are selected as the dominant PCs, which contribute to a large fraction of variances. The ordinary PCA method only focuses on dominant PCs, and ignores the rest of the components, in order to reduce the large dimensionality of the data sets. In most cases, the proportion threshold is set as 80%-90% [3, 4]. In other cases, the researchers cannot find exact implications of the high order PCs, respectively, so they consider them as noise components [4, 5]. PCA pays little attention to the economic meanings of the rest components and just removes the rest components without further analysis [6–8].

However, the remaining components also carry important information. Though the eigenvalues corresponding to the remaining components are small, the huge number of these remaining components makes the sum of their eigenvalues too large to ignore. It is not reasonable to regard the small-eigenvalue components as noise without the analysis of their corresponding eigenvectors. Actually, recent researches find that the remaining components reflect more complicated correlation structure details about financial market, but these components are often covered by the top-ranked components which are identified as the PCs. For example, Plerou et al. (2002) found the business-sector structure using the remaining components of US stock market, and they emphasized the usefulness of the remaining components in construction of optimal portfolios [9]. Sinha and Pan (2007) discovered the industrial correlation structures of the Indian stock market using the rest components [10]. Graczyk and Drarte (2017) studied the trading volume in financial markets, and they found the behavioral homogeneity of the trading volumes [11]. The loss of information embedded in the remaining components is a major limitation of PCA when studying systematic information, but it has received limited attention [1, 2, 6–8].

It is the case especially in the corporate bond market, where the remaining components reveal important information, but only the top-ranked PCs have been concerned [7, 12, 13]. Corporate bond market is one of the most important components in both economy and finance. In China, the corporate bond market is the second largest finance source and even more important to firms than stock markets. The outstanding of net financing of corporate bonds provided by the financial system to the non-financial enterprises and households is 18.59 trillion yuan at the end of February, 2018, which is 2.8 times of the equity financing on the domestic stock market by non-financial enterprises. Besides, the bond market is critically important to the stability of both financial system and macro economy. The global financial crisis from 2008 to 2009 was caused by the corporate bond market in the U.S., which led to a worldwide depression. So it is important to study the corporate bond market, whose behaviors are thought to influence the growth and risks of the economy. The application of PCA to bond markets mainly focuses on the top-ranked PCs. Gilchrist et al. found that the main PCs explain the stable patterns of the corporate bond market [13]. Laurini and Ohashi (2015) applied PCA based on the long-run covariance matrix to forward rate curves and provided a significant reduction in the pricing errors [7]. However, the investors’ considerations, which are dynamic and influenced by the market states, have often been ignored by previous studies, due to the fact that they are contained in the rest components. Though the dominant PCs are well known and studied, the information contained in the remaining components helps the investors to make decisions and win the excess profits. We are inspired to propose a new approach to solve this problem.

In this paper, we propose the General Component Analysis (GCA) based on PCA and the spectrum analysis of large empirical covariance matrices in Bun et.al.(2016) [14]. The complex system could be explained by two types of components, namely global components (GCs) and localized components (LCs). The first step is to identify the two types of components by calculating the inverse participation rates (IPRs). The second step is to analyze the information embedded in the GCs. The third step is to construct the portfolio of the LCs and use the complex network approach to detect its correlation structure. The LCs contain the correlation information about certain bonds, rather than the whole bond market. In other words, the LCs contain cluster information, which reflects the investors’ considerations. The most efficient method to investigate the cluster information in the system is the complex network analysis [15, 16]. The LC portfolio matrix can be treated as an adjacency matrix of the weighted network of bonds, in which the weights indicate how closely the bonds are correlated in their spreads change. Besides, The LC portfolios contain not only useful structure information, but also noise correlations. We use the threshold method to filter the noise correlations. The threshold is derived from shuffled data. The correlation of the shuffled data is considered as noise. We choose the 95th percentile of the shuffled correlations as the threshold in each period. In this way, we filter out most of the noise information contained in the LCs. Through the network analysis, the cluster information contained in the LC portfolios can be discovered. Recent studies about network analysis focus on new methods to construct edges in correlation networks. Tu (2014) studied complex networks based on cointegration rather than correlation the Chinese stock market using [17]. Yan et al. (2016) studied the usage of covariance matrix and correlation matrix in complex system research, and they find that cross-correlation matrices are more suitable to analyze inner correlation structures [18]. The GCA method also extends the application of network analysis.

We apply GCA to the Chinese corporate bond market, and new phenomena are discovered which cannot be found by either PCA or ordinary correlation networks. The rest of the paper is organized as follows: The GCA approach is explained in Section 2. The financial data and time period divisions are discussed in Section 3. The identification results of all the components are shown in Section 4. The GC analysis results are illustrated and discussed in Section 5. The LC analysis results are demonstrated and discussed in Section 6. Finally, we draw our conclusions in section 7.

## General component analysis approach

In this section, we will explain GCA in detail. The daily credit spreads are defined for bond *i* as

$$S_{i}\left(t\right)=y_{i}\left(t\right)-TR_{i}\left(t\right),$$ (1)

where *y*_*i*_(*t*) denotes the valuation yield of corporate bond *i* on day *t*, and *TR*_*i*_(*t*) denotes the valuation yield of the Treasury bond on day *t*, which has the same maturity as the corporate bond *i*. The normalized credit spread for bond *i* is defined as:

$$s_{i}\left(t\right)=\left[S_{i}\left(t\right)-<S_{i}>\right]/\sigma_{i},$$ (2)

where 〈…〉 denotes the average spread over the period studied, and *σ*_*i*_ is the standard deviation of *S*_*i*_(*t*), defined by $\sigma_{i}=\sqrt{<S_{i}^{2}>-<S_{i}{>}^{2}}$. Then, the normalized credit spread matrix is constructed from the time series *s*_*i*_ with the dimensions *N* × *T*:

$$\tilde{S}=[\begin{array}{cccc} s_{11} & s_{12} & \cdots & s_{1T}\\ s_{21} & s_{22} & \cdots & s_{2T}\\ \vdots & \vdots & \ddots & \vdots \\ s_{N1} & s_{N2} & \cdots & s_{NT} \end{array}].$$ (3)

This variable can be used to calculate the *N* × *N* correlation matrix as

$$C=\frac{1}{T}\tilde{S}{\tilde{S}}^{T},$$ (4)

where ${\tilde{S}}^{T}$ is the transpose of $\tilde{S}$. We derivate the linear transformation of the correlation matrix *C* by:

$$C=VDV^{-1}.$$ (5)

$$D=[\begin{array}{cccc} \lambda_{1} & 0 & \cdots & 0\\ 0 & \lambda_{2} & \cdots & 0\\ \vdots & \vdots & \ddots & \vdots \\ 0 & 0 & \cdots & \lambda_{N} \end{array}].$$ (6)

$$V=[\begin{array}{cccc} \nu_{11} & \nu_{12} & \cdots & \nu_{1N}\\ \nu_{21} & \nu_{22} & \cdots & \nu_{2N}\\ \vdots & \vdots & \ddots & \vdots \\ \nu_{N1} & \nu_{N2} & \cdots & \nu_{NN} \end{array}],$$ (7)

where *D* is the diagonal matrix of eigenvalues (λ_1_, λ_2_, …, λ_*N*_) in descending order, and *V* is an orthogonal matrix of the corresponding eigenvectors. Each eigenvalue and the corresponding eigenvector can be written as

$$\lambda_{i}=\nu_{i}^{T}C\nu_{i}=var\left(\nu_{i}^{T}\tilde{S}\right)=var\left(x_{i}\right),$$ (8)

where *ν*_*i*_ is the *i*^*th*^ eigenvector, and $x_{i}=\nu_{i}^{T}\tilde{S}$, which is the *i*^*th*^ component. The eigenvalue λ_*i*_ indicates the variance of component *x*_*i*_. Then, the total variance of the standardized spreads for *N* bonds is

$$tr\left(C\right)=\sum_{i=1}^{N}var\left(x_{i}\right)=\sum_{i=1}^{N}\lambda_{i}=N.$$ (9)

The proportion of total variance in *C* explained by the first *i*^*th*^ component is λ_*i*_/*N*. The ordinary PCA method is a dimension-reduction method, which only explores the economic implications of the first several PCs [19, 20]. Through the PCA method, the components with the largest eigenvalues are selected as the dominant PCs, which contribute to a large fraction of variances. To some extent, the criterion to select the dominant PCs is arbitrary. Most importantly, PCA ignores the remaining components, even though they still carry important structural information.

The GCA provides a comprehensive perspective of all the components, which supplement the PCA. GCA is based on the spectral decomposition of the correlation matrix *C*. When *k* > *R*, the eigenvectors *ν*_*k*_ are meaningless with zero eigenvalues. Other components can be divided into global components (GCs) and localized components (LCs) according to the localization property of the eigenvectors:

$$\begin{array}{c} \begin{array}{cc} C= & VDV^{-1}\\ = & \sum_{k=1}^{R}\lambda_{k}\nu_{k}\nu_{k}^{T}\\ = & \sum_{k\in G}\lambda_{k}\nu_{k}\nu_{k}^{T}+\sum_{k\in L}\lambda_{k}\nu_{k}\nu_{k}^{T}\\ = & C_{GC}+C_{LC}, \end{array} \end{array}$$ (10)

where *G* represents the global component collection, *L* represents the localized component collection and *R* represents the rank of the correlation matrix *C*. GCs are defined as the components which contain systematic information and are analyzed, respectively. LCs are defined as the components which reveal only local structures. It is hard to interpret their implications, respectively, so we combine them into a portfolio.

The GCA approach is comprised of three steps. The first step is the identification of all the components. We use the inverse participation ratio (IPR) to study the localization properties. The IPR of eigenvector is defined as

$$I\left(k\right)=\sum_{l=1}^{N}{\left[\nu_{l,k}\right]}^{4},$$ (11)

where *ν*_*l*,*k*_, *l* = 1, 2, …, *N* is the element of the eigenvector *ν*_*k*_. IPR has been frequently used in physics [21]. The meaning of *I*(*k*) is illustrated by two limiting cases, a vector with identical elements $\nu_{l,k}=\frac{1}{\sqrt{N}},l=1,2,\ldots ,N$ has *I*(*k*) = 1/*N*, whereas a vector with one element *ν*_*k*,1_ = 1 and the remainders zero has *I*(*k*) = 1. Thus, a larger *I*(*k*) indicates that vector *ν*_*k*_ is more localized, and a smaller *I*(*k*) means the vector is global.

In order to provide a natural and data-adjusted reference, rather than an artificial or arbitrary reference, to distinguish the GCs from the LCs, we conduct shuffling operations to the spread data, and take the means of the IPRs calculated from the shuffled data as a reference for identification of GCs and LCs. The shuffled data preserve the distributions of the data series but omit their autocorrelation properties and the physical dependence among different days, which are considered as the random noise contained in the raw data [22].

The shuffling operations are as follows: First, we randomly permute the records in each column of the normalized spread matrix $\tilde{S}$. The permutations of different columns are different so as to omit the dependence among different days. The shuffled normalized spread matrix is noted as ${\tilde{S}}_{shuffled}$. Second, we calculate the correlation matrix from ${\tilde{S}}_{shuffled}$, which is noted as *C*_*shuffled*_. Third, the eigenvectors are derived from *C*_*shuffled*_, and the corresponding IPRs are calculated. We note the shuffled IPR of the *k*_*th*_ component as *I*_*shuffled*_(*k*). During each period, we repeat the operations for 500 times and compute the mean values of the *I*_*shuffled*_(*k*)*s* for each eigenvector. For the *k*_*th*_ component, the mean of the shuffled IPRs is taken as the natural reference to classify GC and LC. If *I*(*k*) derived from the raw data is smaller than the corresponding mean value of *I*_*shuffled*_(*k*)*s*, the *k*_*th*_ component derived from the raw data is identified as a GC, whereas it is identified as an LC. Besides, the shuffled data are also taken as the reference for the modeling choices in the third step.

The second step of GCA is the analysis of the GCs. Usually, the GCs of the system have exact physical or economical implications, so we analyze the global eigenvectors to extract the systematic information.

The third step of GCA is the analysis of the LCs. The economic implications of the separate LCs are obscure and lacking systematic information. It is hard to extract useful information from the separate LCs, so we combine them as a portfolio and apply complex network approach to extract systematic information.

## Data analysis

The key information for our analysis comes from a large sample of corporate bonds issued by Chinese nonfinancial corporations. The size of the Chinese corporate bond market had experienced a dramatic expansion from 2009 to 2014 and reached a steady state after 2014. As we focus on the steady periods to study the influence of the investors’ preferences and macro economy, we analyze the period from January 2014 to April 2016. Since corporate bonds are not transacted in high frequency, the latest transaction price can not reflect the bond’s market price instantly. Therefore, we use the Chinabond valuation, which is widely accepted by regulators and traders, rather than the closed price. The database is comprised of the Chinabond valuation yields of the corporate bonds exchanged in the Chinese inter-bank bond market. The data source is the Wind databases.

We subject our sample to the following conditions: (1) The sample excludes the bonds whose remaining terms to maturity are less than one year, and the included are transacted through the period. (2) The sample excludes the Chinese quasi-municipal bonds according to Wind classification, because the quasi-municipal bonds have implicit backup of the local governments, making yields behave differently to other corporate bonds. (3) The sample excludes imbedded bonds to remove the influence of the imbedded options. (4) We eliminate all observations whose credit spreads are less than 0 basis points or greater than 3500 basis points to mitigate the effect of outliers. In the end, the dataset is comprised of the daily Chinabond valuation of 526 corporate bonds issued by 326 firms covering the period from January 1st, 2014 to April 5th, 2016.

Previews researchers divide time periods according to the peaks and valleys of the index [23], but the index cannot catch the structure breaking situation when some spreads decrease and others increase while the index does not change much. We propose a new method to divide time periods based on the micro data rather than macro index, which performs better in capturing the structure breaking time points. We regard the trading days as observations rather than the spreads, and we use the network method to divide the trading days into different groups. The trading days in the same group have similar spreads behavior, which we called as the same market state. We define the market state as a time period when most of the credit spreads fluctuate around certain values. When the sample covers more than one market states, the correlation structure of spreads changes and the analysis results become unstable. This new time division method is based on the micro data rather than researchers’ judgements, thus it is more reasonable. The construction steps are as follows:

In each trading day, there are 526 bond spreads, which forms a trading day vector. We compute the Euclidean distance of every pair of trading days, and derive the trading day distance matrix *D*, in which the element $D_{t_{x},t_{y}}$ can be calculated as follows:

$$D_{t_{x},t_{y}}=\sqrt{\sum_{i=1}^{N}{\left[S_{i}\left(t_{x}\right)-S_{i}\left(t_{y}\right)\right]}^{2}},$$ (12)

where *S*_*i*_(*t*_*x*_) is the *i*^*th*^ spread on trading day *t*_*x*_, and *S*_*i*_(*t*_*y*_) is the *i*^*th*^ spread on trading day *t*_*y*_. If most of the spreads fluctuate around certain values and do not change dramatically, the distances of the trading days are small, otherwise the fluctuation centers of most spreads change dramatically, the distances are large.

Based on matrix *D*, we construct the trading day network. The nodes represent the trading days. Given a threshold, the nodes are connected by edges when their Euclidean distances are smaller than the threshold.

**Fig 1:**
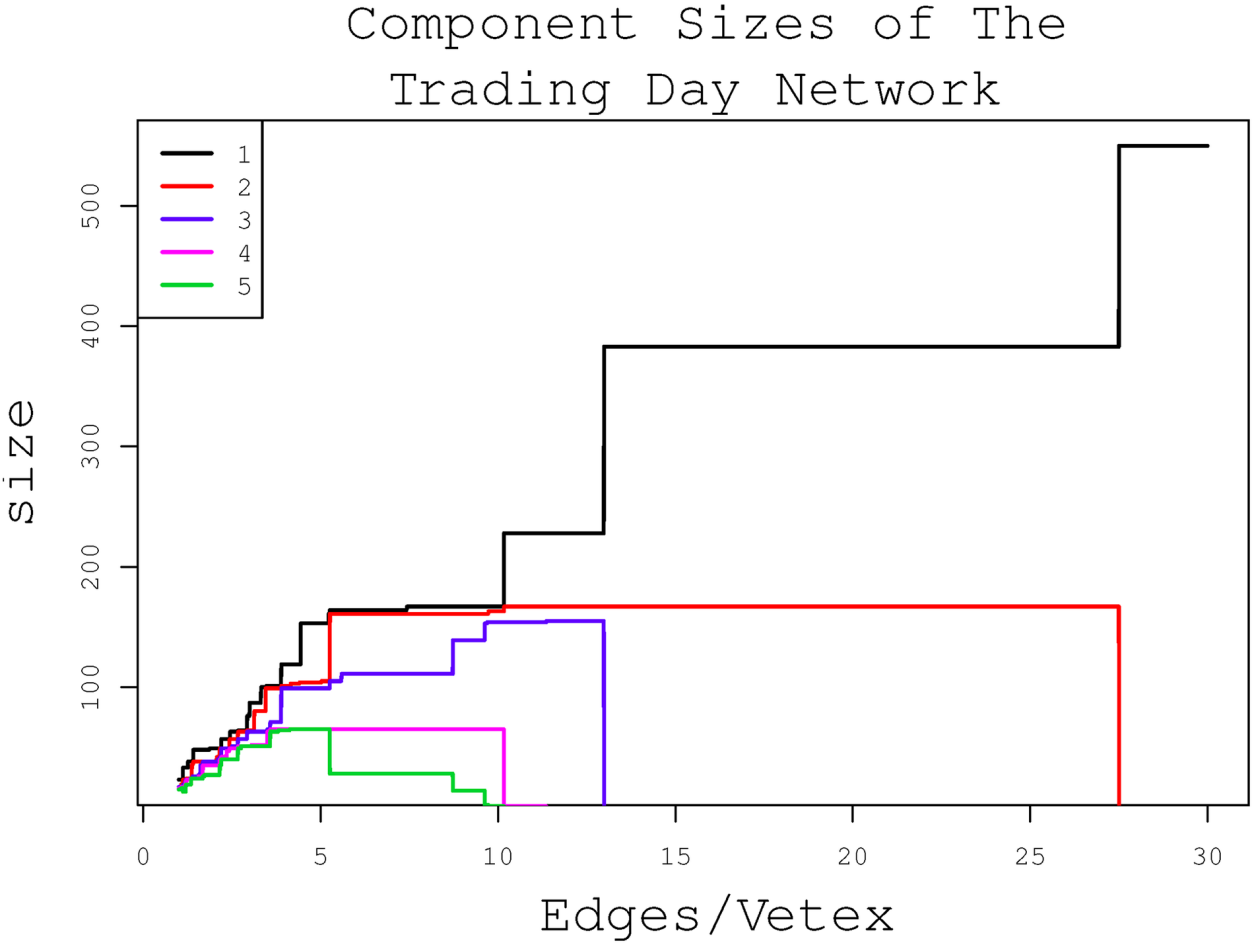
Component sizes of the trading day network. The x-axis represents the total number of edges divided by the sum of vertexes in the trading day network. The y-axis represents the sizes of the 5 largest components in the network. When the sum of the edges is larger than 13 times of the sum of the nodes, the third largest component is connected to the second largest component. Not until the sum of edges increases to 28 times of the sum of the nodes, the second largest component is connected to the largest component.

Fig 1 shows the relation between the sizes of the first 5 largest components in the network and the ratio of the sum of edges to the sum of nodes. When the ratio increases from 0 to 13, the sizes of the components change rapidly. While when the ratio increases from 13 to 28, only two components remain and their sizes do not change. The two stable components indicate that there are two periods in the sample, which is a stable and natural division of periods.

**Fig 2:**
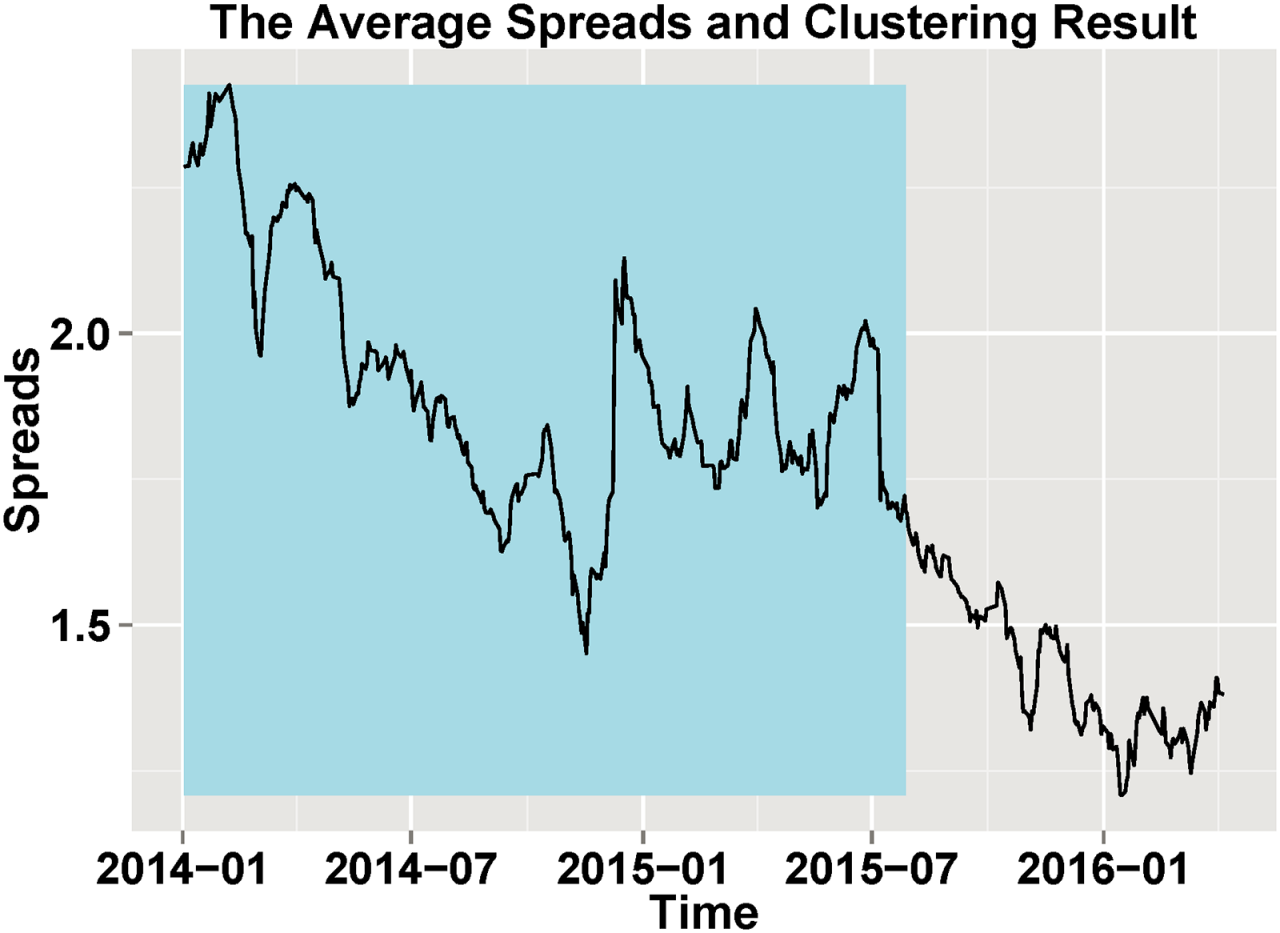
The average spreads and division results. The clustering results are illustrated by shadows, and the line represents the average spreads.

Fig 2 shows the average spreads and division results by shadows. As a result, we use the two periods as the unit of analysis. Period 1 is made up of the trading days from 2014-01-01 to 2015-07-28 while Period 2 is from 2015-07-31 to 2016-04-05. The clustering results guarantee that the correlation structure stays the same, which is critical for the analysis in the next sections.

## The identification results of all the components

In this section, we will first analyze the localization properties of the eigenvectors to identify the global components, localized components and noise components.

**Fig 3:**
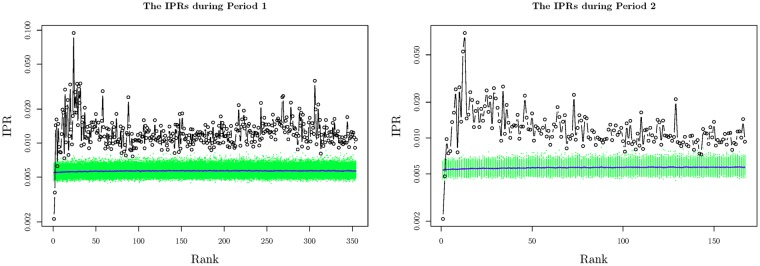
The IPRs of the eigenvectors. The x-axis represents the rank of eigenvectors *ν*_*k*_. The y-axis represents the corresponding IPR *I*(*k*). The black circles and lines represent the *I*(*k*)s of the eigenvectors *ν*_*k*_ derived from the spreads set $\tilde{S}$. The green points represent the *I*(*k*)s of the eigenvectors derived from the shuffled data. The blue solid lines represents the mean values of the *I*(*k*)s derived from the shuffled data.

We calculate IPRs of the eigenvectors through the two periods, respectively. The results are shown in Fig 3. The IPRs *I*(*k*) reach a peak when *k* = *R*. When *k* > *R*, the eigenvectors *ν*_*k*_s are identified as noise components. During each period, we conduct the shuffling operations mentioned in Section 2 for 500 times. The shuffled IPRs are also shown in Fig 3 by green points, which are distributed uniformly around 0.06 for the first *R* eigenvectors in both periods.

As a result, the first 2 components are identified as the GCs in both periods, while the 3^*rd*^ to the 384^*th*^ (*R*^*th*^) components in Period 1 and the 3*rd* to the 168^*th*^ (*R*^*th*^) components in Period 2 are the LCs. The 2 GCs explain 88.09% and 85.44% of the variance in spreads during the 2 periods, respectively, while the 382 LCs in Period 1 and 166 LCs in Period 2 explain 11.91% and 14.56% of the variance. It is unreasonable to ignore the LCs.

## The global component analysis results

Then we look into the eigenvectors of the GCs to find out the interpretations. We use the box plots to illustrate the distributions of the eigenvectors, respectively, during the two periods. According to the credit risk theory, especially the reduced form model [24], the credit risks mainly depends on the terms to maturity and bond ratings [25, 26]. Therefore, the box plots demonstrate the relationship between the eigenvectors’ distributions and terms to maturity or ratings, respectively.

**Fig 4:**
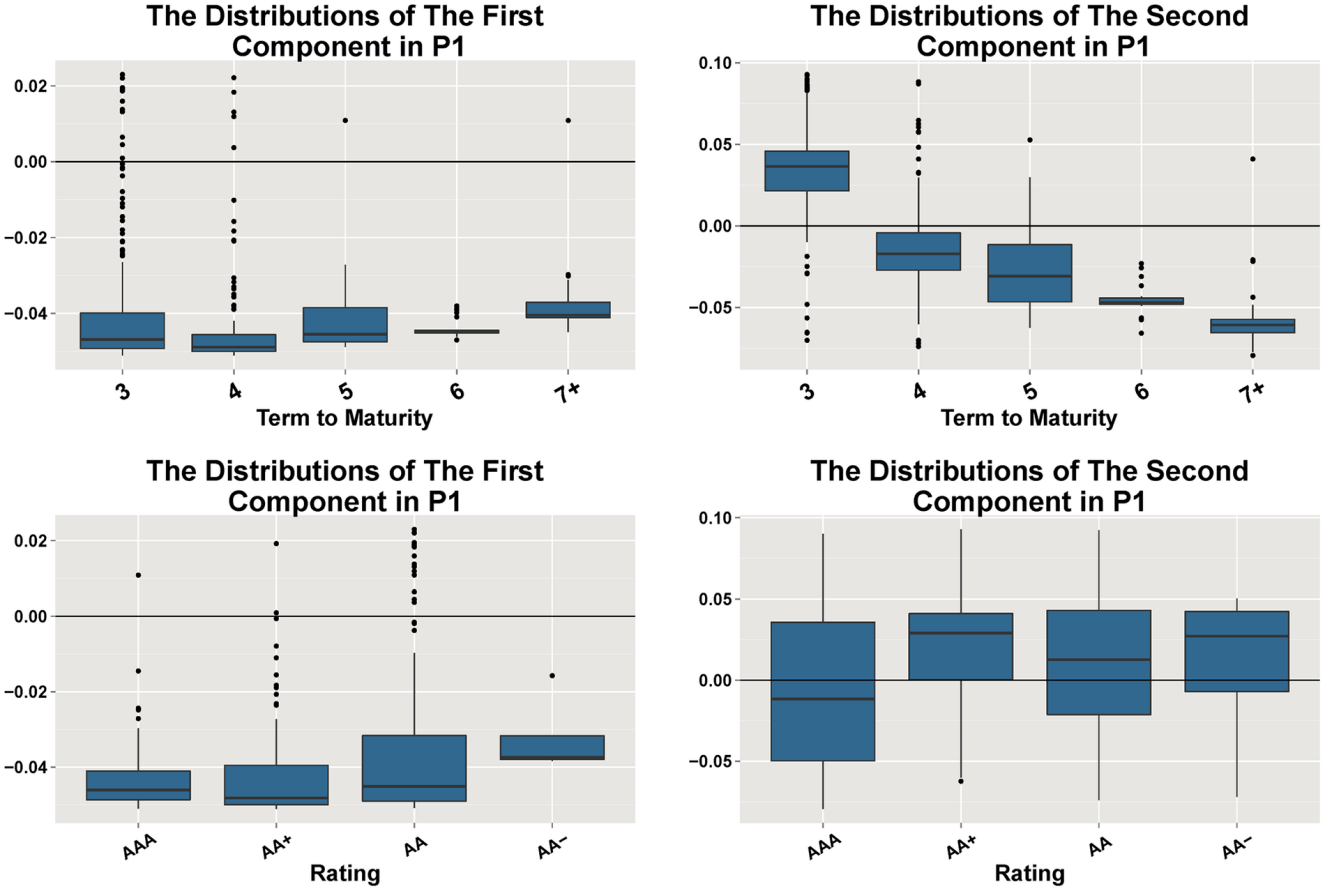
The eigenvectors’ distributions of the two GCs in Period 1. The box plots in the first row illustrate the distributions of the two GCs grouped by terms to maturity, respectively, in Period 1 (from 2014-01-02 to 2015-07-28). We use “7+” to denote the terms longer than 7. The box plots in the second row illustrate the distributions grouped by ratings. The second GC is relevant to terms to maturity, while neither component is relevant to ratings.

Fig 4 illustrates the distributions of the two GCs grouped by terms to maturity and ratings in Period 1, respectively. All of the coefficients of the first GC are negative except a few outliers, which means that the spreads move in the same direction. In the second GC, the box plot shows that longer terms to maturity lead to lower distributions of the coefficients, which means the long term bonds move in the opposite direction with the short term bonds.

**Fig 5:**
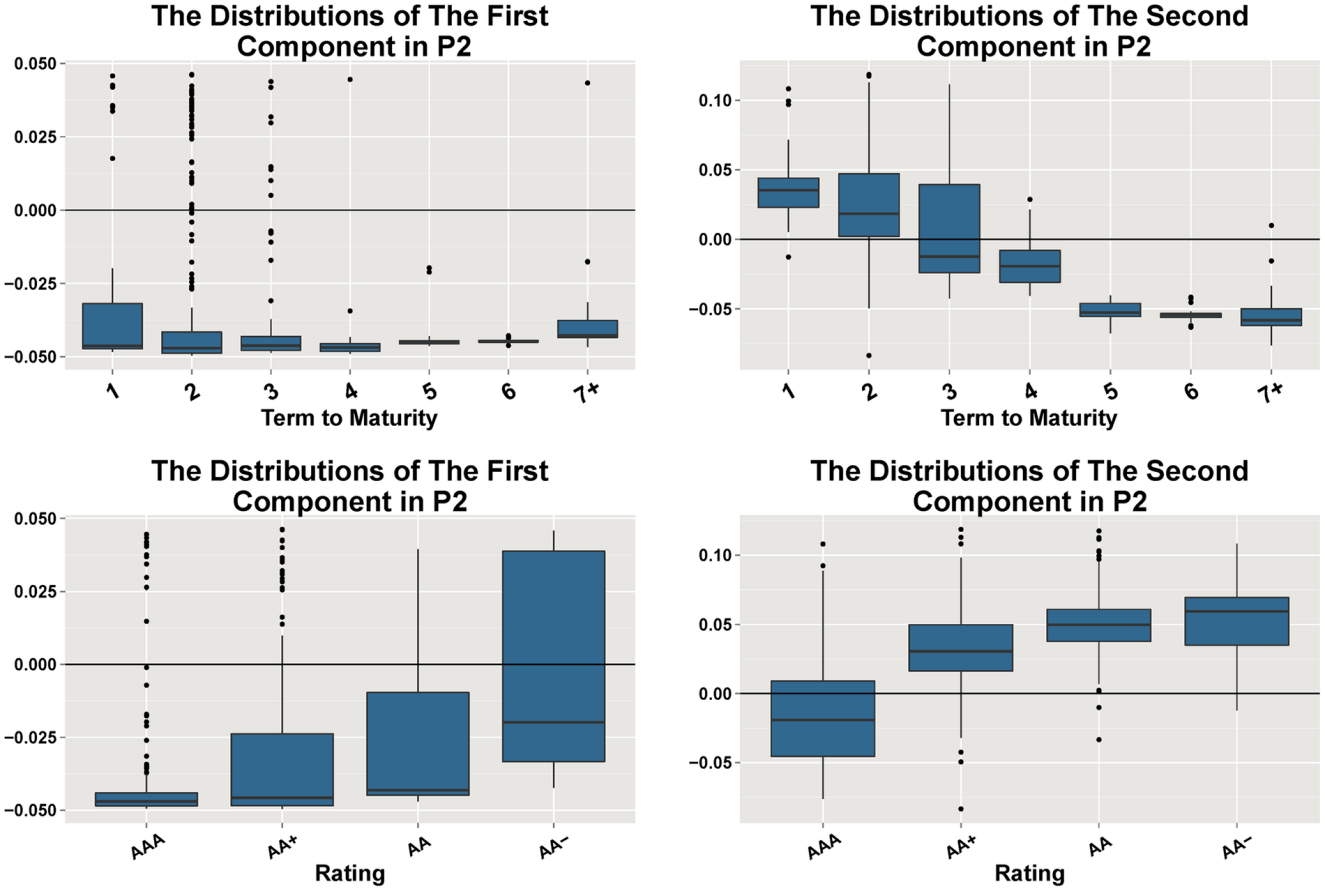
The eigenvectors’ distributions of the two GCs in Period 2. The box plots in the first row illustrate the distributions of the two GCs grouped by terms to maturity, respectively, in Period 2 (from 2015-07-28 to 2016-04-05). While the second row are the distributions grouped by ratings.

Fig 5 shows the interpretations of the two GCs during Period 2, which are consistent with those of Period 1. The coefficients of the first GC are negative, which reflects co-movements. Besides, the first GC is also relevant to ratings. The bonds with lower ratings have higher dispersions in the first GC distribution. That is, the lower the bond ratings are, the more dispersed its coefficient distributions become. The second GC is relevant not only to terms to maturity, but also bond ratings. In the second PC, the lower the ratings are, the higher the coefficient distributions are. It means the spreads of high rated bonds (mainly AAA-rated bonds) move in different direction with the spreads of low rated bonds (mainly AA and AA- rated bonds). The reason why both of the GCs in Period 2 are relevant to ratings is that many default events broke out then due to the declining increasing rate of macro economy, and the investors managed to sell the low-rated bonds.

## The localized component analysis results

Besides the global components, LCs are also very important in describing market characteristics. The economic meanings of the separate LCs are obscure and chaotic. It is hard to extract meaningful information from separate LCs, especially the higher number of components, using the same way as PCA. Besides, there are hundreds of LCs in the complex system, and it is impossible and unnecessary to study all the LCs separately. So We construct the LC portfolios by the following formula, and apply network analysis to identify the cluster information:

$$C_{LC}=\sum_{k=q}^{R}\lambda_{k}\nu_{k}\nu_{k}^{T}.$$ (13)

Recall the discussions in the previous section, *q* = 3, *R* = 384 in Period 1, and *q* = 3, *R* = 168 in Period 2. The reason why we use the weighted summation of LCs rather than the equal-weighted summation is that we would like to include the eigenvalue information into consideration. The eigenvectors contain the structural information of the system, and the eigenvalues indicate the influence power of the components to the system. It is necessary to combine both of the eigenvalue and eigenvector information.

**Fig 6:**
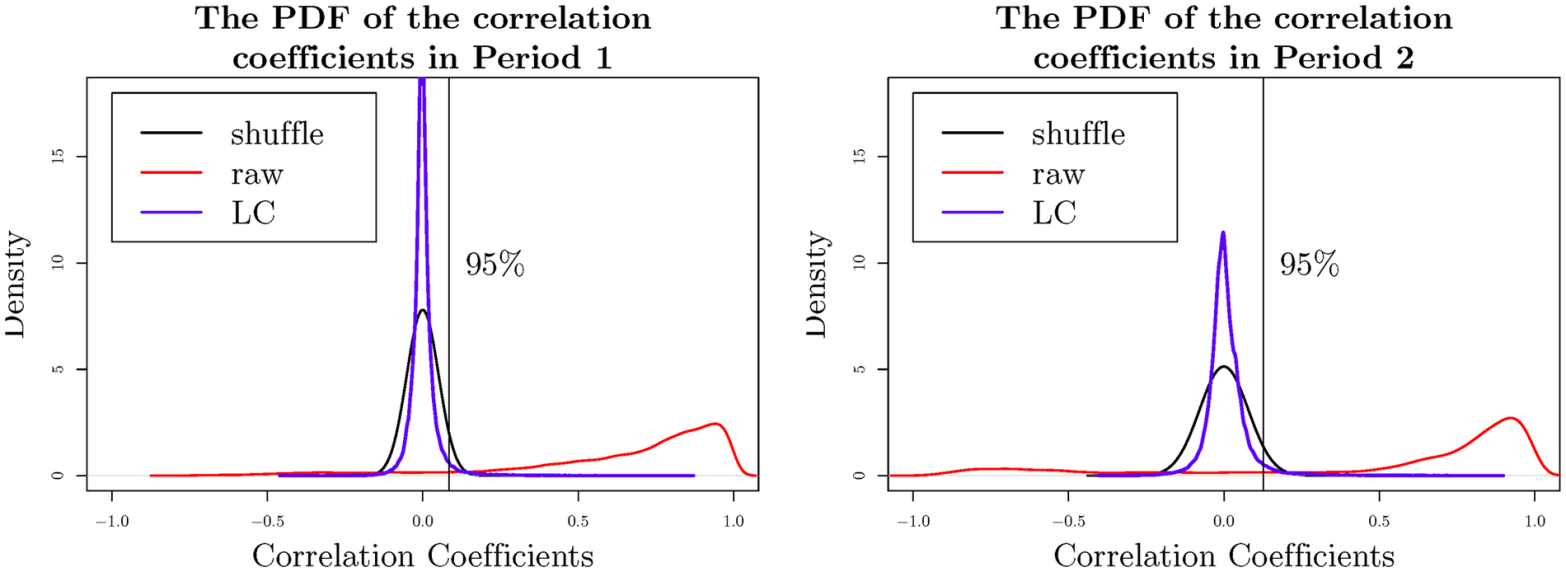
The PDFs of the shuffled correlations, the raw-data correlations and the LC portfolio elements. The black line represents the probability density function (PDF) of the shuffled correlation coefficients. The red line represents the PDF of the correlation coefficients derived from the raw data. The blue line represents the PDF of the coefficients of *C*_*LC*_.

In order to demonstrate the results clearly, we construct networks based on *C*_*LC*_ by the threshold method. The LC portfolios contain not only useful structure information, but also noise correlation. As a proper threshold should be able to filter the noise links in the network, it inspires us to select the thresholds based on the shuffled data *C*_*shuffle*_ derived in Section 4. The correlations of the shuffled data (*C*_*shuffle*_) are considered as the noise correlations, and we choose the 95th percentile of the shuffled correlations as the threshold in each period. The threshold is 0.0842 in Period 1, and 0.1274 in Period 2. Fig 6 shows the PDFs of the shuffled correlations, the raw-data correlations and the LC portfolio elements.

We construct networks by filtering the edges according to their weights. Only the edges whose weights are higher than the threshold can be kept.

We define the weighted degree in the same way as Barrat et. al. (2004), $WD_{i}=\sum_{j=1}^{N}a_{ij}w_{ij}$, where *a*_*ij*_ is the element of the adjacent matrix, and *w*_*ij*_ is the weight of the edge connecting vertex *i* to vertex *j*. WD measures the strength of a vertex in terms of the total weights of all its connections [27]. The larger the *WD* is, the more important the vertex is to the system.

We apply the Louvain method to the LC networks to detect clusters. The Louvain method is a heuristic method based on modularity optimization and outperforms other methods in terms of computation time and modularity quality [28]. In order to interpret the clusters derived from the LC networks, we define the weighted degree proportion (WDP) of an economic property as

$$WDP\left(P_{l},C_{j}\right)=\frac{\sum_{i=1}^{N}\delta_{i|P_{l}}\delta_{i|C_{j}}WD_{i}}{\sum_{i=1}^{N}\delta_{i|C_{j}}WD_{i}}$$ (14)

$$\delta_{i|P_{l}}=\{\begin{array}{ccc} 1, & & if\;vertex\;i^{\prime }s\;economic\;property\;is\;P_{l},\\ 0, & & otherwise. \end{array}$$ (15)

$$\delta_{i|C_{j}}=\{\begin{array}{ccc} 1, & & if\;vertex\;i\;belongs\;to\;cluster\;C_{j},\\ 0, & & otherwise. \end{array}$$ (16)

where *P*_*l*_ denotes the *l*^*th*^ type of the economic property *P*, such as the Central State-Owned Enterprise (CSOE) (*P*_*L*_) for the ownership type(*P*), and *C*_*j*_ denotes the *j*^*th*^ cluster.

The *WDP*(*P*_*l*_, *C*_*j*_) measures the importance of economic property *P*_*l*_ to cluster *C*_*j*_ by the sum of the WDs of the nodes satisfying both *C*_*j*_ and *P*_*l*_ divided by that of the nodes in *C*_*j*_. If *WDP*(*P*_*l*_, *C*_*j*_) is greater than 50%, we consider cluster *C*_*j*_ to be mainly influenced by property *P*_*l*_.

We compute the *WDPs* of enterprise ownerships, ratings and overcapacity industries (OCIs). These three properties are considered to have great influence on the spreads. There are three types of enterprise ownerships, i.e. Central State-Owned Enterprise (CSOE), local state-owned enterprises (LSOE) and private enterprises (PE). There are four levels of bond ratings, i.e. AAA, AA+, AA and AA-. The OCI bonds are defined as the bonds whose issuers belong to iron or mining industry, because Chinese government put iron and mining industries on the list of overcapacity industries (OCIs) in February 2016. The developments of the OCIs were to be constrained by the government, which was an important step of the supply-side reform in China and its impact on the corporate bond market was significant. Additionally, we compute the average term to maturity of each bond cluster to help us interpret the networks.

### The LC analysis results of Period 1

**Fig 7:**
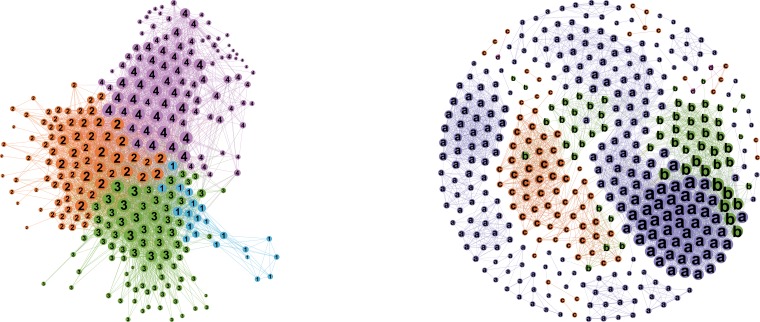
The ordinary correlation network and the LC network of spreads during Period 1. The left plot shows the LC network of spreads in Period 1. The colors and the numbers on the nodes denote which clusters the nodes belong to. The sizes of the vertices are measured by WDs. The right plot shows the ordinary correlation network of spreads in Period 1, whose nodes cluster according to ratings. The characters and colors denote the credit ratings of the bonds, where a denotes AAA, b denotes AA+, c denotes AA, and d denotes AA-.

In this part, we will present the LC analysis results of Period 1. The left plot of Fig 7 demonstrates the LC network during Period 1. The sum of the edges in the LC network is 4108, which is 7.81 times of the sum of vertexes. The Louvain method is applied to the network and 4 clusters are identified. The WDPs of ownership, rating and OCI, and the average term to maturity of every cluster are computed to help us to understand the network. All of the results are demonstrated in Table 1.

**Table 1:** The clusters’ WDPs of ownership, rating and OCI, as well as the average term during Period 1.

	WDP of Ownerships			WDP of Ratings					
Clusters	CSOE	LSOE	PE	AAA	AA+	AA	AA-	WDP of OCI	average term
1	**67.30%**	32.70%	0.00%	**100.00%**	0.00%	0.00%	0.00%	11.53%	**5.00**
2	37.38%	30.01%	32.61%	44.77%	17.03%	38.20%	0.00%	22.39%	2.37
3	33.23%	31.80%	34.97%	**53.50%**	14.42%	32.08%	0.00%	30.70%	3.53
4	3.18%	48.77%	48.05%	9.59%	15.20%	**67.26%**	7.96%	18.95%	1.45
Total	24.15%	37.75%	38.10%	35.43%	15.08%	46.45%	3.04%	22.95%	2.57

In the LC network, the spreads form 4 clusters in Period 1. Among them, most of the bonds in Cluster 1 are long-term, AAA-rated bonds. These bonds are mainly issued by critical CSOEs who play important roles in the development of the Chinese national economy, such as the China National Nuclear Corporation and the China Three Gorges Corporation. It will threaten the safety of Chinese economy and national defense if these CSOEs default and go bankrupt. The investors consider these bonds to have the implicit governmental guarantees so that their chances to default are much lower. That is why they are often called the super AAA bonds by the investors. Cluster 2 is similar to Cluster 3 in respect to the ownerships and ratings, but the average term of Cluster 3 is longer. Cluster 4 is mainly comprised of the PE bonds and LSOE bonds. Because the credit risk of PE bonds is higher than the LSOE and CSOE bonds, Cluster 4 includes more AA-rated bonds.

The right plot of Fig 7 shows the ordinary correlation network of spreads during Period 1 to demonstrate the advantage of the LC network. We tried to apply the same threshold as the LC network, and then the ordinary correlation network includes 125813 edges, which is 239.18 times of the sum of nodes. The ordinary correlation network is too dense to discover the correlation structures. So we keep the same number of the edges as the LC network, which is 7.81 times of the sum of nodes. We find that the clusters are divided according to the bond ratings. Though rating is an important factor for spread pricing according to the reduced form approach, spreads are influenced by many factors, and they change with the economic states. This is where the advantage of LC networks comes in, as it sheds light on other factors which are usually ignored such as ownership as well.

### The LC analysis results of Period 2

Finally, we demonstrate the LC analysis results of Period 2. The sum of the edges in the LC network is 5112, which is 9.72 times of the sum of vertexes. The Louvain method is applied to the network and 4 clusters are identified. The *WDPs* of ownership, rating and OCI, as well as the average term to maturity are computed and demonstrated in Table 2.

**Table 2:** The clusters’ WDPs of ownership, rating and OCI, as well as the average term during Period 2.

	WDP of Ownerships			WDP of Ratings					
Clusters	CSOE	LSOE	PE	AAA	AA+	AA	AA-	WDP of OCI	average term
1	**67.26%**	28.41%	4.34%	**92.21%**	6.15%	1.64%	0.00%	7.88%	**5.58**
2	39.99%	37.22%	22.79%	42.68%	45.73%	8.19%	3.40%	16.96%	2.45
3	20.20%	49.86%	29.94%	31.44%	28.12%	35.15%	5.29%	**61.22%**	2.41
4	28.98%	44.24%	26.78%	44.57%	16.01%	38.99%	0.42%	27.53%	3.01
Total	27.58%	45.55%	26.88%	39.41%	26.32%	30.81%	3.45%	43.29%	3.26

**Fig 8:**
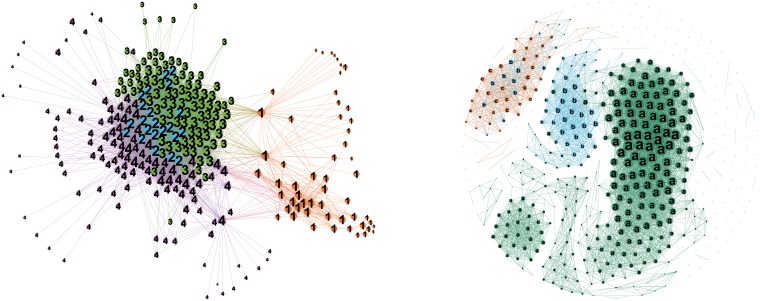
The ordinary correlation network and the LC network of spreads during Period 2. The left plot is the LC network of spread in Period 2 and the right plot is the ordinary correlation network of spreads in Period 2. The denotations of these plots are the same as Fig 7.

The left plot of Fig 8 demonstrates the LC network during Period 2. Cluster 1 is the collection of super AAA bonds with long term to maturity. This cluster has been consistently identified in both of the periods. Cluster 3 is comprised of bonds whose issuers belong to OCIs, whose WDP of OCI is 61.22%. Since the Chinese government was planning the supply-side reform, the OCIs were expected to reduce production by merging and acquisition, and the firms in these industries were encouraged to deleverage. The bonds issued by firms of these industries were facing huge default risk, though the average spread of the whole market was declining. The ratings of bonds in Cluster 3 range from AAA to AA-. The diversification of the ratings shows that investors would like to sell the OCI bonds no matter what levels of their ratings were. However, China Shenhua Energy Company Limited, which is the biggest mining corporation and a CSOE, is not included in this cluster, due to the fact that it is of vital importance to the Chinese economic development and can also obtain implicit governmental guarantee. Other bonds are split into two clusters, i.e. cluster 2 and cluster 4. Cluster 2 includes more AA+ rated bonds while Cluster 4 includes more AA rated bonds. The two clusters are connected to each other, which indicates that most of the bond spreads fluctuate following the main part of the market. This fact is widely accepted by the bond market participants.

## Discussions

We compare the GCA results with that of ordinary correlation network analysis at first. The right plot of Fig 8 shows the ordinary correlation network of spreads during Period 2. If We use the same threshold as the LC network, the ordinary correlation network will include 109286 edges, which is 207.76 times of the sum of nodes. It is too dense to discover the inner correlation structure. So we keep the same number of the edges as the LC network, which is 9.72 times of the sum of nodes. In the ordinary correlation network, the clusters are still divided according to ratings, the same as Period 1.

Throughout the two periods, the clustering results of the ordinary correlation networks reflect only the ratings of the bonds, without information related to macro-economic events or important policies. The results of the LC networks perform better than the correlation network approach in this regard as it shall reflect the dynamic evolution of market and economic state. In addition, it shall identify at least two collections of bonds worth attention. One cluster is comprised of the super AAA bonds, which are the long-term bonds issued by the important CSOEs with less default risk. Investors considered these bonds to have implicit governmental guarantees, and they tended to buy these bonds if the expectation of default was high. The other cluster is comprised of the OCI bonds, due to the fact that investors tended to sell the OCI bonds because of the supply-side reform.

We also compare the GCA results with that of PCA. PCA selects the dominant PCs by the proportion that the PCs contribute to the total variance, which is arbitrary in some extent. We set the proportion threshold to be 90%, which is widely used in previous studies [3, 29, 30]. We identify the first 3 components in Period 1 (which contribute to 91.16% total variance) and the first 3 components in Period 2 (which contribute to 91.67% total variance) as the dominant PCs.

**Fig 9:**
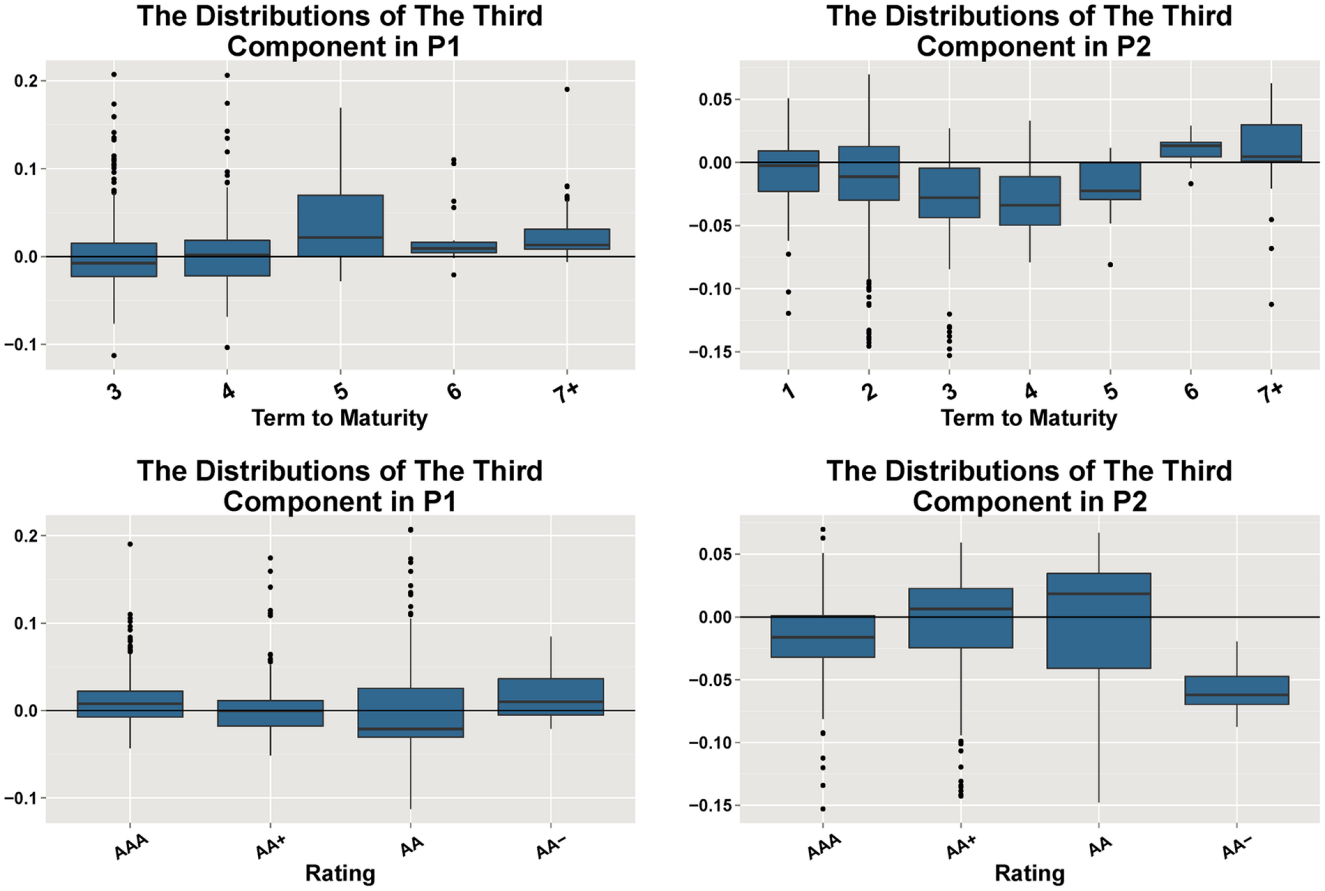
The box plots illustrate the distributions of the coefficients of the third components in Period 1 and Period 2. In the first row, the distributions are grouped by terms to maturity, and in the second row the distributions are grouped by ratings. The third components in both periods are relevant to neither terms to maturity nor ratings.

Recall that we have analyzed the first two PCs in Section 5. The first PC reflects the co-movements of the spreads. The second PC reflects the impact of terms to maturity and ratings, which can be seen in Figs 4 and 5. The third components in the two periods are relevant to neither terms nor ratings, which are shown in Fig 9. The third components in the two periods are relevant to neither terms nor ratings.

Compared with PCA, GCA extracts the economic meanings contained in the top two PCs, which are the main discoveries of PCA. Moreover, GCA extracts more complicated and useful information of the spread systems by the analysis of LCs, which are ignored by PCA. In this study, GCA discovers that the super AAA bond collection behaves different from other bonds due to the implied governmental guarantees. Besides, GCA also discovers the co-movements of OCI bond spreads in Period 2 due to the impact of supply-side reform. PCA fails to extract these useful information such as the super AAA bonds and OCI bonds, which are ignored in the components with smaller contributions to variances while identified by our new GCA method.

All in all, we would not have been able to identify the super AAA bond collection and the OCI bond collection without our new method.

## Conclusions

In this work, we are inspired to propose General Component Analysis (GCA) to extract the systematic information from all of the components. Both of the GCs and LCs are identified according to their localization property. The mean value of IPRs derived from the shuffled data is defined as the identification threshold, which is a natural and exquisite reference. This natural identification guarantees the stableness of later analysis. By contrast, PCA selects dominant PCs only by the eigenvalues while ignores the information of the eigenvectors. It throws out many components at higher orders, which are mainly the LCs. By the analysis of the LC portfolio, it is interesting and instructive to find out the structure information concealed by the GCs and ignored by PCA.

Using the Chinese corporate bond market as an example, we demonstrate the advantages of GCA. We propose a new network based method to divide time series, which is better at identifying the time points when the market state switches. Using this method, two incompatible periods can be found in the given data set. Among them, Period 2 was caused by the supply-side reform in this case. This step is important in guaranteeing the stable correlation structures in further analysis.

GCA results are as follows: first, there are two GCs in the spreads system. The results of GC analysis show that the first GC reflects the market co-movement while the second GC is relevant to terms to maturity. In Period 2, the two GCs are correlated to credit ratings due to the high default risk. Second, there are 382 LCs in Period 1 and 166 LCs in Period 2. Two interesting collections can be extracted from the LC portfolios, which are helpful to understand the thoughts of the investors. One is the super AAA bond collections which is believed to have implicit governmental guarantees by the investors in both periods, and the other is the overcapacity industrial bond collection which is influenced by the supply-side reform led by the Chinese government in Period 2. These novel and interesting phenomena reveal a deeper structure of the system, which cannot be identified by either the ordinary correlation network method or PCA. GCA is expected to be applied to other complex systems, such as the stock market [31, 32], to draw a complete picture of them.
